# Mitochondrial Dynamics Mediated by DRP1 and MFN2 Contributes to Cisplatin Chemoresistance in Human Ovarian Cancer SKOV3 cells

**DOI:** 10.7150/jca.61379

**Published:** 2021-10-28

**Authors:** Guang-Ping Zou, Chun-Xia Yu, Sheng-Lan Shi, Qiu-Gen Li, Xiao-Hua Wang, Xin-Hui Qu, Zhang-Jian Yang, Wei-Rong Yao, Dan-Dan Yan, Li-Ping Jiang, Yu-Ying Wan, Xiao-Jian Han

**Affiliations:** 1 Institute of Geriatrics, Jiangxi Provincial People's Hospital Affiliated to Nanchang University, Nanchang, Jiangxi 330006, P.R. China.; 2 Department of Intra-hospital Infection Management, the Second Affiliated Hospital of Nanchang University, Nanchang, Jiangxi 330006, P.R. China.; 3 Department of Neurology, Jiangxi Provincial People's Hospital Affiliated to Nanchang University, Nanchang, Jiangxi 330006, P.R. China.; 4 Research Institute of Ophthalmology and Visual Sciences, Affiliated Eye Hospital of Nanchang University, Nanchang, Jiangxi 330006, P.R. China.; 5 Department of Pharmacology, School of Pharmaceutical Science, Nanchang University, Nanchang, Jiangxi 330006, P.R. China.; 6 Department of Oncology, Jiangxi Provincial People's Hospital Affiliated to Nanchang University, Nanchang, Jiangxi 330006, P.R. China.

**Keywords:** ovarian cancer, mitochondrial dynamics, chemoresistance, cisplatin, apoptosis

## Abstract

Cisplatin (DDP) is the first-line chemotherapeutic agent for ovarian cancer. However, the development of DDP resistance seriously influences the chemotherapeutic effect and prognosis of ovarian cancer. It was reported that DDP can directly impinge on the mitochondria and activate the intrinsic apoptotic pathway. Herein, the role of mitochondrial dynamics in DDP chemoresistance in human ovarian cancer SKOV3 cells was investigated. In DDP-resistant SKOV3/DDP cells, mitochondrial fission protein DRP1 was down-regulated, while mitochondrial fusion protein MFN2 was up-regulated. In accordance with the expression of DRP1 and MFN2, the average mitochondrial length was significantly increased in SKOV3/DDP cells. In DDP-sensitive parental SKOV3 cells, downregulation of DRP1 and upregulation of mitochondrial fusion proteins including MFN1,2 and OPA1 occurred at day 2~6 under cisplatin stress. Knockdown of DRP1 or overexpression of MFN2 promoted the resistance of SKOV3 cells to cisplatin. Intriguingly, weaker migration capability and lower ATP level were detected in SKOV3/DDP cells. Respective knockdown of DRP1 in parental SKOV3 cells or MFN2 in SKOV3/DDP cells using siRNA efficiently reversed mitochondrial dynamics, migration capability and ATP level. Moreover, MFN2 siRNA significantly aggravated the DDP-induced ROS production, mitochondrial membrane potential disruption, expression of pro-apoptotic protein BAX and Cleaved Caspase-3/9 in SKOV3/DDP cells. In contrast, DRP1 siRNA alleviated DDP-induced ROS production, mitochondrial membrane potential disruption, expression of pro-apoptotic protein BAX and Cleaved Caspase-3/9 in SKOV3 cells. Thus, these results indicate that mitochondrial dynamics mediated by DRP1 and MFN2 contributes to the development of DDP resistance in ovarian cancer cells, and will also provide a new strategy to prevent chemoresistance in ovarian cancer by targeting mitochondrial dynamics.

## Introduction

Ovarian cancer is one of the most common gynecological cancer in the female reproductive system, and becomes the second leading cause of death in gynecological cancer in the world 1. It is estimated that 230, 000 women will be diagnosed with ovarian cancer and 150, 000 will die per year. At the early stage of ovarian cancer, most patients have no obvious symptoms, thus leading to an absence of effective early diagnosis. Nowadays, ideal cytoreductive surgery and neoadjuvant chemotherapy are regarded as the standard treatment for ovarian cancer, and the combination of Cisplatin (DDP) and Paclitaxel is the primary chemotherapy 2. Although ovarian cancer is sensitive to DDP at the beginning of treatment, the chemoresistance to DDP is gradually developed in most patients when the treatment period is prolonged 3. In general, the uptake of cisplatin into cells mainly depends on two kinds of transporters, including copper influx transporter (CTR) and organic cation transporter (OCT) 4, 5. In cancer cells, DDP damages DNA and finally causes apoptosis. Pieces of evidence indicates the efflux of intracellular DDP, the reduction of cytotoxic cisplatin-DNA adducts, the enhanced ability of DNA repair 6, 7, and inhibition of pro-apoptotic pathways contribute to the development of DDP chemoresistance. As a result, DDP chemoresistance leads to the recurrence of ovarian cancer and a five-year overall survival rate of less than 30% 8. On the other hand, the intracellular DDP can directly impinge on the mitochondria, decrease ATP synthases activity and induce the mitochondrial apoptotic pathway in cancer cells 9-11. Therefore, it is necessary to investigate whether mitochondrial dynamics is involved in the development of DDP chemoresistance.

Mitochondria are the important power plants that provide the most ATP for the activities of cells. In addition, mitochondria are also highly dynamic organelles with constant movement in cells, and their morphology varies through frequent fusion and fission events, namely mitochondrial dynamics 12, 13. In mammalian cells, mitochondrial fission is mediated by DRP1 and mitochondrial adaptor fission 1 (Fis1), while the fusion of outer and inner mitochondrial membrane is regulated by mitofusins (MFN1, MFN2) and optic atrophy 1 (OPA1), respectively 13, 14. It is well documented that mitochondrial dynamics participates in various cellular activities, such as oxidative stress 15-17, apoptosis 18-20, mitophagy 20-22, even nutrient utilization and energy consumption 23. Mitochondrial dynamics are also important for the maintenance of mitochondrial normal shape, quantity and function 22, 24. It was reported that the abnormal mitochondrial dynamics is related to the tumorigenesis 25. Excessive mitochondrial fission induces intracellular ROS production, disruption of mitochondrial membrane potential, and eventually leads to cellular damage or death. In our previous studies, upregulation of DRP1 was found to facilitate the hypoxia-induced migration of breast cancer MDA-MB‑231 cells, and sensitized cancer cells to DDP chemotherapy 26. DRP1 inhibitor Mdivi-1 effectively attenuates DDP-induced intracellular ROS, caspase-3 activation and cell death in murine leukemia L1210 cells 27. Furthermore, miR-148a-3p promotes mitochondrial fission, and enhances the sensitivity of gastric cancer to cisplatin 28. Overexpressing N-Myc in neuroblastoma cells alleviates the DDP-induced cell death through increasing mitochondrial fusion 29. However, the role of mitochondrial dynamics in cisplatin chemoresistance in ovarian cancer and its underlying mechanism remains to be elucidated.

In the present study, we first examined the expression of mitochondrial dynamics-related proteins in SKOV3 and SKOV3/DDP cells. It was found that mitochondrial fission protein DRP1 was downregulated in SKOV3/DDP cells, while mitochondrial fusion protein MFN2 was upregulated. Compared to SKOV3 cells, the average length of mitochondria was also increased in SKOV3/DDP cells. Moreover, knockdown of DRP1 or overexpression of MFN2 in SKOV3 cells promoted its resistance to cisplatin. Intriguingly, weaker migration capability and lower ATP level were detected in SKOV3/DDP cells. Respective knockdown of DRP1 in SKOV3 or MFN2 in SKOV3/DDP cells using siRNA reversed mitochondrial dynamics, migration capability and ATP level. In addition, MFN2 siRNA aggravated the DDP-induced ROS production, mitochondrial membrane potential disruption, expression of pro-apoptotic protein BAX and Cleaved Caspase-3/9 in SKOV3/DDP cells. In contrast, DRP1 siRNA alleviated DDP-induced ROS production, mitochondrial membrane potential disruption, expression of pro-apoptotic protein BAX and Cleaved Caspase-3/9 in SKOV3 cells. Therefore, our data indicate mitochondrial dynamics mediated by DRP1 and MFN2 may play a critical role in the development of DDP chemoresistance in ovarian cancer.

## Materials and Methods

### Cell culture

Two ovarian cancer cell lines, SKOV3 (obtained from ATCC) and SKOV3/DDP Cells (purchased from the Tumor cell bank of the Chinese Academy of Medical Science) were used in this study. SKOV3 and SKOV3/DDP were separately cultured in Dulbecco's Modified Eagle's medium (DMEM, Invitrogen, USA) or RPMI-1640(Invitrogen, USA) supplemented with 10% fetal bovine serum (Invitrogen, Carlsbad, CA, USA), 1% streptomycin and 1% ampicillin. All cells were maintained in a humidified incubator at 37℃ with an atmosphere containing 5% CO_2_. In the experiments of cisplatin stress, SKOV3 cells were exposed to 1.00 mg/L cisplatin from day 0~6, and the culture medium was replaced with fresh medium containing the same concentration of cisplatin once every three days.

### Cell viability

SKOV3 and SKOV3/DDP cells were seeded in 96-well plates (5 × 10^3^ cells per well). After treatment with DDP for 24 hours, cell viability was detected by cell counting kit-8 (CCK-8, APExBIO, USA). Briefly, the mixture of CCK-8 test solution (10 μL) and culture medium (100 μL) was added into each well. After incubation for 2 h at 37℃ in the dark, the absorbance (OD value) at 450 nm was measured to evaluate the cell viability using a microplate reader (Multiskan Mk3, Thermo Scientific, USA). Cells incubated with normal culture medium were used as control. The wells with only medium were set as blank. Cell viability was calculated according to the following formula: [OD(DDP)-OD(Blank)]/[OD(Control)-OD(Blank)] × 100%.

### Western blot

Cells were washed three times with Phosphate-buffered saline (PBS) and were lysed in radioimmunoprecipitation assay (RIPA) lysis buffer containing protease inhibitors. The whole cell lysate of SKOV3 or SKOV3/DDP cells was collected, and was further subjected to sonication. The total protein concentration of cell lysate was measured with a bicinchoninic acid (BCA) protein assay kit (#23227, Thermo, USA). Proteins in lysate were separated by 10% SDS-poly-acrylamide gels electrophoresis and then transferred on PVDF membrane (HATF00010, Millipore, Billerica, MA). After blocked with 5% bovine serum albumin for 1 h at room temperature, the interest protein on PVDF membranes was immunoblotted overnight with following primary antibodies: DRP1 Rabbit mAb (1:1000; #8570, Cell signal technology, USA), Mitofusin-2 Rabbit mAb (1:1000; #9482S, Cell signal technology, USA), OPA1(1:1000; ab42364, Abcam, USA), Mitofusin-1 Rabbit mAb (1:1000; #D6E2S, Cell signal technology, USA), Cleaved Caspase-9 antibody (1:1000; #20750, Cell signal technology, USA), Cleaved Caspase-3 antibody (1:1000; #9644, Cell signal technology, USA), GAPDH antibody (1:2000; sc-25778, Zhongshan Jinqiao, China), BAX antibody(1:1000; #5023, Cell signal technology, USA) , β-actin (1:1000; AP0060, Bioworld Technology). After washed three times with TBST, the membranes were further incubated with HRP-conjugated secondary antibody (1:5000; CW0103T, Cwbiotech, China) for 1 h at room temperature. The bands were captured by using an enhanced chemiluminescence solution (K-12045-D10, Advansta, USA). Finally, the densitometric analysis was performed using ImageJ software. Chemiluminescence assay was performed with Amersham Enhanced Chemiluminescence Prime Western Blotting Detection reagents (CWBIO, Beijing, China). The immunoblot signal was detected using the Molecular Imager®Chemi DOCTXRS+ system (Bio-Rad Laboratories, Inc., Hercules, CA, USA) and the density of each band was measured using ImageJ software (National Institutes of Health, Bethesda, MD, USA).

### RNA interference

Small interference RNA was used for knockdown of the DRP1 and MFN2 gene in SKOV3 or SKOV3/DDP cells. For silencing DRP1 expression, the sequences of siRNA targeted DRP1 and its scramble are the same as our previous reported 26. DRP1 siRNA sequence: 5′-GAGUUAUGAACGACUCAdTdT-3′ (forward) and 5′- TGAGGCGTCAATAACCTCdTdT-3′ (reverse); scramble siRNA: 5′-UUCUCCGAACGUGUCACGUCACGUdT-3′ (forward); 5′-ACGUCACGUCGGAAdT-3′ (reverse); MFN2 siRNA-1 sequence: 5′- CCAAAUUGCUCAGGAAUAATT-3′ (forward) and 5′-UUAUUCCUGAGCAAUUUGGTT-3′ (reverse); MFN2 siRNA-2 sequence: 5'-AGGTGCTCAACGCCAGGATTC-3' (forward) and 5'-AGTCGGTCTTGCCGCTCTTCA-3' (reverse); MFN2 siRNA-3 sequence: 5′-GGAGAUUGAGGAGUGCAUdTdT-3′ (forward) and 5′-AUGCACACCUCAAUCUCCdTdTdT-3′ (reverse); Scramble RNA: 5′-UUCUCCGAACGUGUCACGUCACGUdT-3′ (forward), 5′-ACGUGACACGUGAAdTdT-3′ (reverse). The optimal concentration of DRP1 siRNA and MFN2 siRNA are separately 10 nmol/L and 20 nmol/L. For RNA interference, DRP1 siRNA and MFN2 siRNA were separately transfected into SKOV3 and SKOV3/DDP cells using lipofectamine2000 according to the manufacturer's instructions. In brief, cells were seeded in a 6-well culture plate. The mixture of corresponding siRNA and transfection reagent was added into the culture well when the confluence of SKOV3 or SKOV3/DDP cells reach 70%~80%. The culture medium was replaced with growth medium at 6 h after transfection, and the silencing efficiency of DRP1 or MFN2 was evaluated using western blotting assay at 48 h after transfection. Cells transfected with scramble RNA were used as negative control (NC).

### Plasmid transfection

The full length cDNAs of human MFN2 were obtained by RT-PCR using total isolated RNA from SKOV3 cells, and subcloned into the mammalian expression plasmid pcDNA3.1 (Invitrogen). The pcDNA3.1-MFN2 plasmid was confirmed by sequencing. The empty vectors and MFN2 plasmid were transfected into SKOV3 cells using lipofectamine 2000 reagent (Invitrogen, Carlsbad, CA) according to the manufacturer's instructions. The infection medium containing lipofectamine 2000 was replaced by growth medium at 6 h after transfection.

### Mitochondrial imaging and analysis

To label mitochondria, SKOV3 and SKOV3/DDP cells were transfected with pDsRed2-Mito. The transfection procedure was conducted according to the manufacturer's instructions of Lipofectamine 2000 (Invitrogen) as described previously 30. At 24 h after transfection, mitochondrial morphology was detected under a Zeiss confocal microscope (LSM 800; Carl Zeiss Microscopy, Germany) by visualizing the fluorescent signal of DsRed2 with excitation at 545nm. The mitochondrial length was measured and analyzed using ZEN 2.3SP1. For each group, approximately 270 mitochondria from four different random fields were measured.

### Detection of intracellular ROS level

The intracellular ROS level in SKOV3 and SKOV3/DDP cells was measured using the fluorescent probe 2',7'-dichlorodihydrofluorescein diacetate assay kit (DCFH-DA, Sigma-Aldrich, USA) according to the manufacturer's instructions. Briefly, SKOV3 or SKOV3/DDP cells were incubated with 10 μM of DCFH-DA for 30 min at 37℃ in the dark after treatment with or without DDP (4mg/L) for 24 h. Then, cells were washed three times with PBS and harvested. The mean fluorescence intensity was detected as intracellular ROS level using flow cytometry (DxFLEX, Beckman Coulter Biotechnology) with the excitation at 488 nm and emission at 525 nm. Data analysis was carried out using GraphPad Prism 9.0 (GraphPad Software, Inc., La Jolla, CA).

### Measurement of mitochondrial membrane potential (Δψm)

Tetramethylrhodamine methyl ester (TMRE, Invitrogen) staining was used to detect the mitochondrial membrane potential (Δψm) as described previously 30. In brief, cells were incubated with 100 nM TMRE for 20 min in darkness at 37 ℃ after treatment with or without 4 mg/L DDP (purchased from Sigma, USA) for 24 h. Then cells were washed twice with PBS, and mean fluorescence intensity of TMRE in cells were detected as mitochondrial membrane potential using flow cytometry (DxFLEX, Beckman Coulter Biotechnology) with the excitation at 543 nm and emission at 560 nm. Data analysis was carried out using GraphPad Prism 9.0 (GraphPad Software, Inc., La Jolla, CA).

### Measurement of intracellular ATP level

The intracellular ATP level was detected in the lysate of SKOV3 or SKOV3/DDP cells using the enhanced ATP assay system bioluminescence Detection Kit (Promega, Madison, WI, USA). Cells were harvested and lysed in a 2% trichloroacetic acid and 2 mM EDTA solution at 48 h after RNA interference. The lysate was subjected to a 12000×g centrifuge at 4 ℃ for 5 min, the supernatant was used for intracellular ATP assay. According to the manufacturer's instructions, 20 μl supernatant in each sample and ATP test solution were co-incubated at room temperature for 3-5 min, then ATP level was measured using a microplate reader (Multiskan Mk3, Thermo Scientific, USA). Then, the ATP level was normalized to the protein content in each sample (indicated as nmol/mg of protein).

### Transwell assay

After transfected with DRP1 siRNA and MFN2 siRNA for 24 hours, cells were trypsinized and reseeded on the upper chamber at a density of 2 × 10^ 4^ cells in 200 μl of the medium. The lower chamber contained 500 μl complete medium with 10% FBS. 24 h later, the cells on the upper chamber were gently removed with a cotton swab. The migrated cells on the nether surface were fixed with 4% PFA for 10 min at room temperature and stained with crystal violet for 30 min. The migrated cells were further photographed under a microscope, and counted in at least three random fields. All assays were independently repeated at least three times.

### Wound healing assay

The wound healing assay was performed as the previously reported 31. In brief, cells were seeded on 35-mm wells, and cultured in the complete growth medium. Then, the monolayer of cells was scratched with a constant width by a 200-μl pipette tip. After a scratch, cells were washed twice with PBS to remove the suspended cells and further cultured in a medium without FBS. The wound closure was photographed at 0 h, 24 h, 48 h after scratch, and the wound healing percentage was measured using GraphPad Prism 9.0 (GraphPad Software, Inc., La Jolla, CA). The wound healing percentage = (0 hour's width-N hours' width)/ (0 hour's width) * 100%, N=24 or 48.

### Statistical analysis

All data are shown as the mean ± SD. Data were analyzed using either t-test to compare two conditions or ANOVA to compare multiple conditions, and *p*<0.05 was considered to be significant.

## Results

### The mitochondrial dynamics mediated by DRP1 and MFN2 are different between SKOV3 and SKOV3/DDP cells

**Figure 1:**
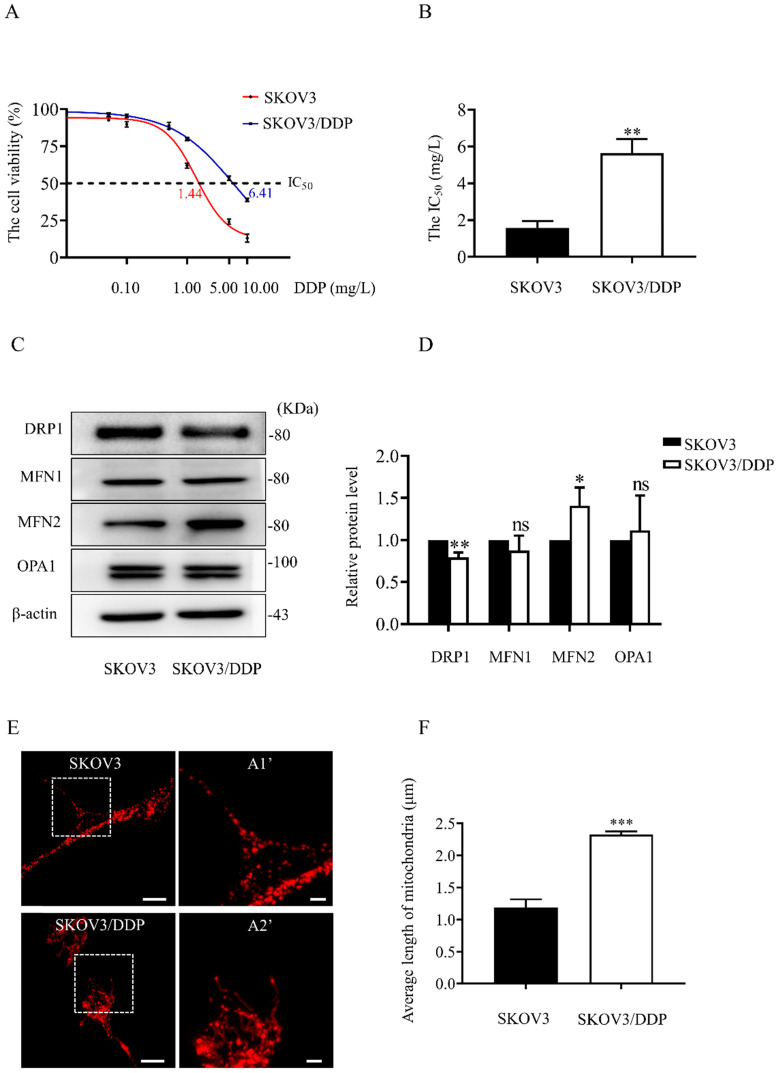
** The expression of mitochondrial dynamics related proteins in SKOV3 and SKOV3/DDP cells**. (A) The cisplatin dose-response curve in SKOV3 and SKOV3/DDP cells. Two cell lines were exposed to DDP at different concentrations (0.01, 0.05, 0.1, 0.5, 1, 5, 10 mg/L) for 24 hours. Cell viability was determined by CCK-8 assay kit. (B) The IC50 of cisplatin against SKOV3 and SKOV3/DDP cells was calculated according to the cisplatin dose-response curve. (C) The western immunoblotting of DRP1, MFN1, 2 and OPA1 in SKOV3 and SKOV3/DDP cells. The total proteins in whole cell lysate of two cell lines were separated by SDS-PAGE electrophoresis, and the expression levels of DRP1, MFN1, MFN2, and OPA1 were detected using western blotting assay. β-actin was used as the endogenous reference. (D) The densitometric analysis of DRP1, MFN1, MFN2, and OPA1 in (C) was performed from three independent experiments. The relative expression level of each protein is indicated as a normalization of the ratio of mitochondrial dynamics-related protein/β-actin in each sample to the control. (E) Mitochondrial morphology in SKOV3 and SKOV3/DDP cells. The pDsRed2-Mito was transferred into SKOV3 and SKOV3/DDP cells to label mitochondria. The red fluorescence signal of DsRed was detected as mitochondrial morphology in cells under a confocal microscope, scale bar=20 μm. A1' and A2' show mitochondria with higher magnification in the inserted boxes, scale bar = 5 μm. (F) The difference in average length of mitochondria between SKOV3 and SKOV3/DDP cells. The length of 270 mitochondria in each group was measured and the average length was calculated from three independent experiments. * p < 0.05, ** p < 0.01, *** p < 0.001 (SKOV3 vs. SKOV3/DDP). Abbreviations: DDP, cisplatin.

**Figure 2:**
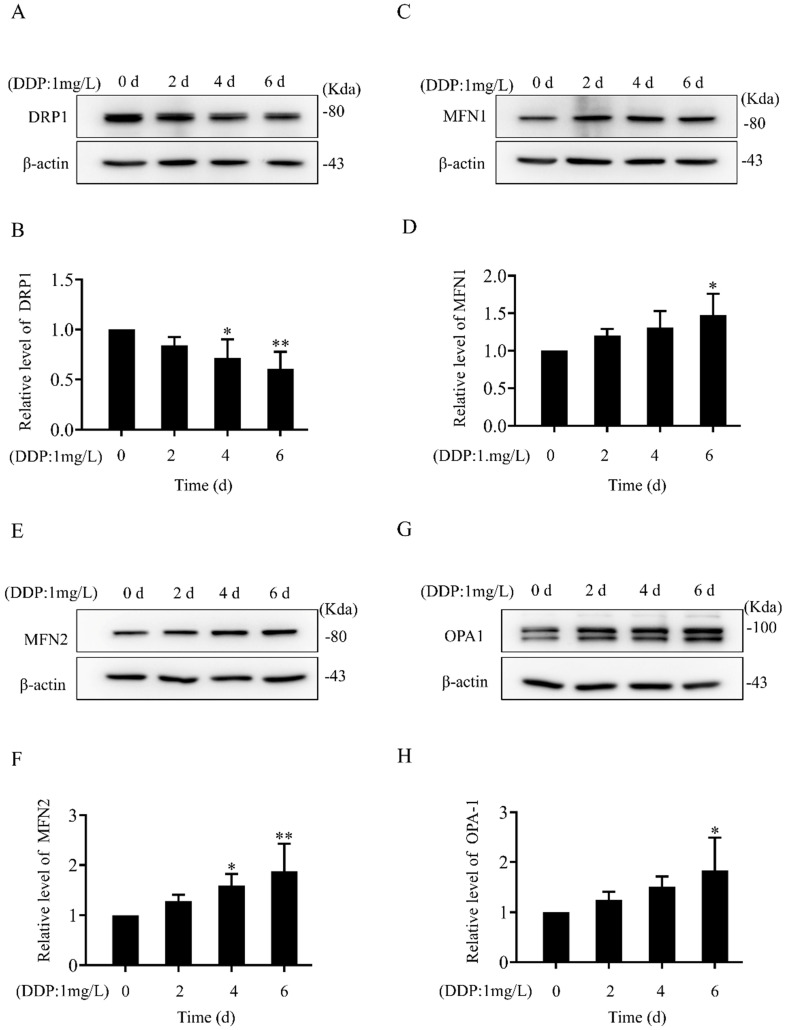
** Alteration in the expression of mitochondrial dynamics-related proteins in SKOV3 cells under cisplatin stress.** (A, C, E, G) The western immunoblotting of mitochondrial dynamics-related proteins in SKOV3 cells under cisplatin stress. The whole cell lysates of SKOV3 cells treated with 1mg/L cisplatin for 0 d, 2 d, 4 d, and 6 d were separated by SDS-PAGE electrophoresis, and the expression of DRP1, MFN1, MFN2 and OPA1 were detected using western blotting assay. β-actin was used as the endogenous reference. (B, D, F, H) The densitometric analysis of DRP1, MFN1, MFN2, and OPA1 in (A, C, E, G) was performed from three independent experiments. The relative expression level of each protein is indicated as a normalization of the ratio of mitochondrial dynamics-related protein/β-actin in each sample to the control. *p < 0.05, **p < 0.01 vs. 0 d.

**Figure 3:**
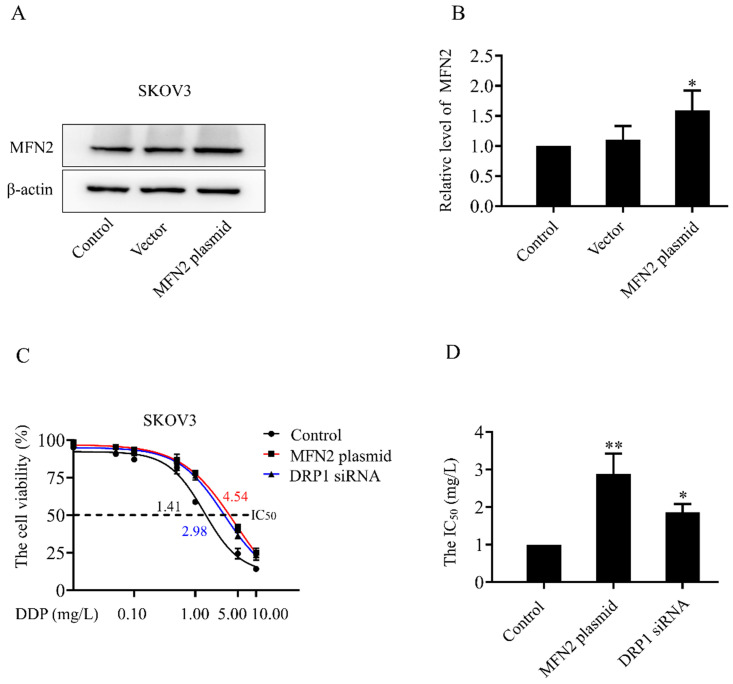
** The sensitivity of SKOV3 cells to DDP after knockdown of DRP1 or overexpression of MFN2.** (A) The expression level of MFN2 in SKOV3 cells transfected with MFN2 plasmid was detected using western blotting assay. β-actin was used as the endogenous reference. (B) The densitometric analysis of MFN2 in (A) was performed from three independent experiments. The relative expression level of MFN2 is indicated as a normalization of the ratio of MFN2/β-actin in each sample to the control. (C) After transfected with DRP1 siRNA for 48 h or MFN2 plasmid for 24 h, SKOV3 cells were exposed to DDP at different concentrations (0, 0.01, 0.05, 0.1, 0.5, 1, 5, 10 mg/L) for 24 hours. Cell viability was determined by CCK-8 assay kit. (D) The IC50 of cisplatin against SKOV3 cells was calculated according to the cisplatin dose-response curve. *p < 0.05, **p < 0.01 vs. Control.

**Figure 4:**
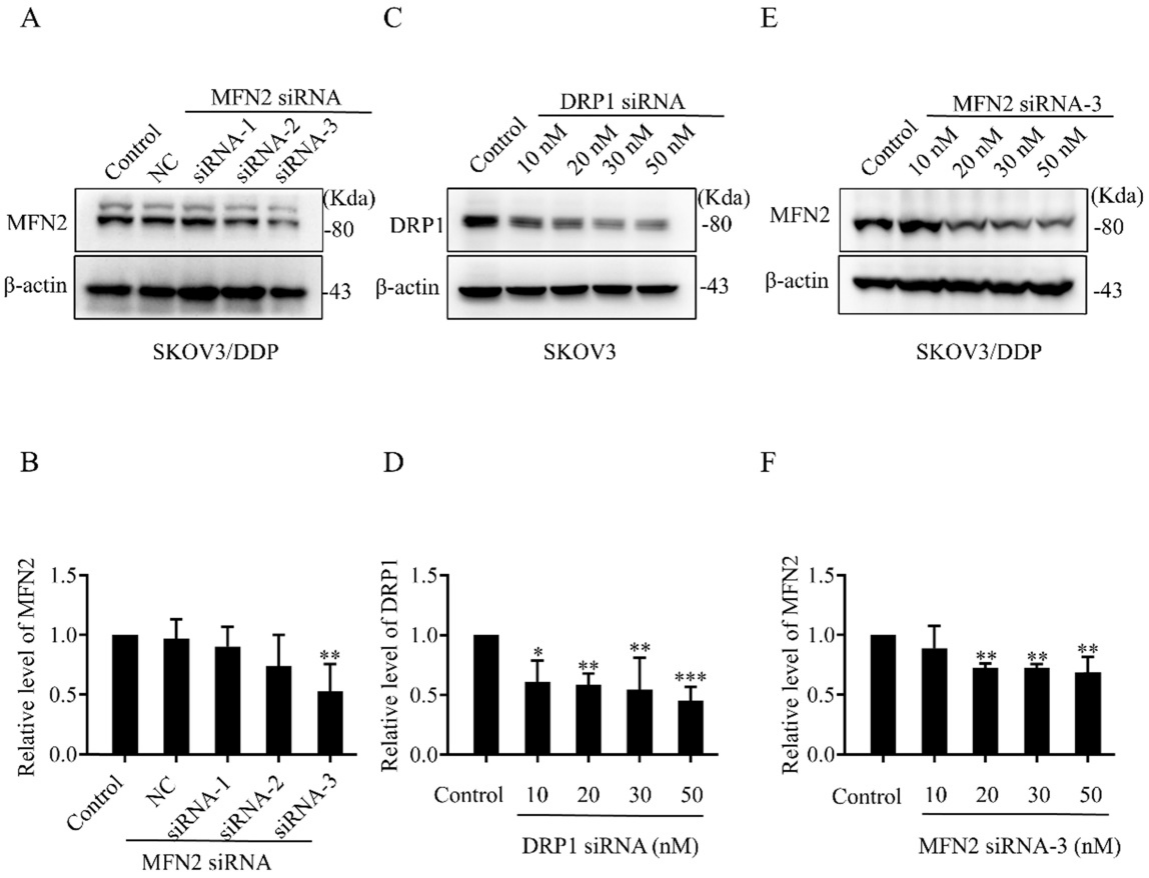
**The silencing efficiency of DRP1 and MFN2 in SKOV3 and SKOV3/DDP cells by siRNA.** (A, C, E) The expression of DRP1 and MFN2 in SKOV3 and SKOV3/DDP cells transfected with DRP1 siRNA and MFN2 siRNA was detected using western blotting assay. β-actin was used as the endogenous reference. (B, D, F) The densitometric analysis of DRP1 and MFN2 in (A, C, E) was performed from at least three independent experiments. The relative expression level of each protein is indicated as a normalization of the ratio of interest protein/β-actin in each sample to the control. * p < 0.05, ** p < 0.01, *** p < 0.001 vs. Control.

In both SKOV3 and SKOV3/DDP cells, treatment with DDP for 24 h reduced the cell viability in a dose-dependent manner (Fig. 1A). In addition, the IC_50_ of DDP against SKOV3 cells and SKOV3/DDP cells was 1.44 mg/L and 6.41 mg/L, respectively (Fig. 1B). These results indicate that SKOV3/DDP cells are more resistant to DDP than SKOV3 cells. To examine the role of mitochondrial dynamics in DDP resistance in ovarian cancer cells, we first examined the expression of mitochondrial dynamics-related proteins including MFN1, MFN2, DRP1, and OPA1 in SKOV3 and SKOV3/DDP cells. Compared to SKOV3, the expression of mitochondrial fission protein DRP1 was downregulated, while mitochondrial fusion protein MFN2 was upregulated in SKOV3/DDP cells (Fig. 1C and 1D). Next, pDsRed2-Mito was transfected to SKOV3 and SKOV3/DDP cells to label mitochondria. Consistent with the expression level of DRP1 and MFN2, mitochondria appeared as elongated tubular network structures in SKOV3/DDP cells (Fig. 1E). The average mitochondrial length in SKOV3/DDP was significantly longer than that in SKOV3 cells (Fig. 1F). The differential expression of DRP1 and MFN2 between SKOV3 and SKOV3/DDP cells suggests its possible role in the development of DDP chemoresistance. To explore the underlying mechanism, the expression of mitochondrial dynamics related proteins was further examined in SKOV3 cells at day 0~6 under DDP stress. As shown in Fig. 2, DRP1 was downregulated in SKOV3 cells at day 4 and day 6 after treated with 1mg/L DDP. In contrast, mitochondrial fusion protein MFN2 was upregulated at day 4~6 after DDP treatment, MFN1 and OPA1were upregulated at day 6 after DDP. To examine the role of DRP1- or MFN2-mediated mitochondrial dynamics in the chemoresistance of SKOV3 cells to DDP, knockdown of DRP1 by siRNA or overexpression of MFN2 were conducted in SKOV3 cells (Fig. 3A, 3B and Fig. 4C). Intriguingly, knockdown of DRP1 or overexpression of MFN2 significantly increased the IC50 of DDP against SKOV3 cells (Fig.3C and 3D). These results suggest that mitochondrial fusion mediated by upregulated MFN2 and downregulated DRP1 might facilitate the development of DDP chemoresistance in ovarian cancer.

### Knockdown of DRP1 or MFN2 using siRNA changes mitochondrial morphology and ATP production in SKOV3 or SKOV3/DDP cells

**Figure 5:**
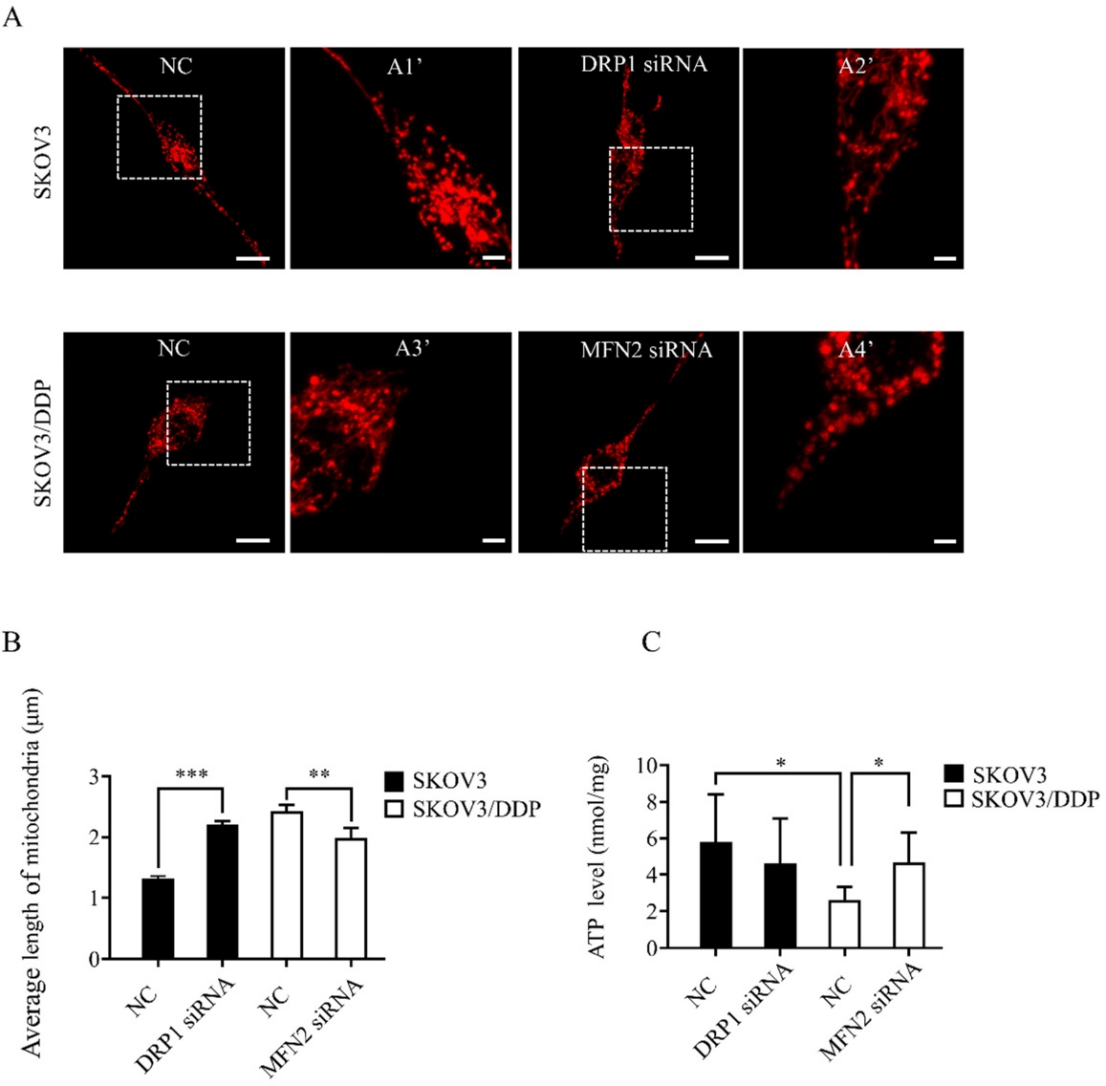
**Knockdown of DRP1 or MFN2 changed mitochondrial morphology and ATP level in SKOV3 or SKOV3/DDP cells.** (A) Mitochondrial morphology in SKOV3 or SKOV3/DDP cells transfected with or without the related siRNA. pDsRed2-Mito was transferred into SKOV3 and SKOV3/DDP cells. The fluorescence signal of pDsRed2-Mito was detected as mitochondrial morphology in cells under a confocal microscope, scale bar=20 μm. A1', A2', A3' and A4' show mitochondria with higher magnification in the inserted boxes, scale bar = 5 μm. (B) The length of 270 mitochondria in each group was measured and the average length was calculated from three independent experiments. (C) The intracellular ATP level in SKOV3 and SKOV3/DDP cells transfected with or without the targeting siRNA. At 48 h after transfection, the intracellular ATP level in two cell lines was detected using a commercial ATP assay kit under the microplate reader, and the ATP level was normalized to the protein content in each sample. * p < 0.05, * p < 0.01, *** p < 0. 001.

To explore the role of mitochondrial dynamics mediated by DRP1 and MFN2 in DDP chemoresistance, silencing DRP1 and MFN2 using siRNA was conducted in SKOV3 and SKOV3/DDP cells, respectively. The siRNA used for silencing DRP1 is the same as reported in our previous study 2. To silence MFN2, three siRNAs targeting MFN2 and a scramble siRNA were synthesized. As shown in Fig. 4C and 4D, 10~50 nM of siRNA efficiently decreased the expression of DRP1 in SKOV3 cells to approximately 40%~50%. Thus, 10 nM of DRP1 siRNA was used in the following experiments. In addition, the silencing efficiency of siRNA-1~3 targeting MFN2 was examined in SKOV3/DDP cells. It was found that the silencing efficiency of siRNA-3 targeting MFN2 was the best (Fig. 4A and 4B). The silencing efficiency of siRNA-3 in different concentrations was also evaluated. As shown in Fig. 4E and 4F, 20~50 nM of siRNA-3 had a similar silencing efficiency on MFN2. Therefore, 20 nM of siRNA-3 was used for silencing MFN2 in SKOV3/DDP cells in the following experiments. Next, we further examined the effect of DRP1 siRNA and MFN2 siRNA on mitochondrial morphology in SKOV3 and SKOV3/DDP cells, respectively. As expected, mitochondrial morphology appeared in elongated tubules, the average length was significantly increased in SKOV3 cells after silencing DRP1. In contrast, silencing MFN2 in SKOV3/DDP cells shifted mitochondria from tubules to the fragmented or punctate dot-like shape, the average length of mitochondria was significantly decreased (Fig. 5). In addition, mitochondria are the main energy plants for ATP generation in mammalian cells 32, 33. Given the different mitochondrial dynamics mediated by DRP1 and MFN2 between SKOV3 and SKOV3/DDP cells, we measured the ATP level in two cell lines. Unexpectedly, the ATP level was much lower in SKOV3/DDP cells than that in SKOV3 cells. Although silencing DRP1 had no significant effect on the ATP level in SKOV3 cells, silencing MFN2 dramatically increased the ATP level in SKOV3/DDP cells (Fig. 5C). According to the results above, silencing DRP1 and MFN2 by siRNA provides a reliable means to manipulate mitochondrial dynamics in SKOV3 and SKOV3/DDP cells, and it is also important for exploring the role of mitochondrial dynamics mediated by DRP1 and MFN2 in the development of DDP chemoresistance in ovarian cancer.

### Mitochondrial dynamics mediated by DRP1 and MFN2 is involved in mitochondrial membrane potential and ROS production in SKOV3 and SKOV3/DDP cells

**Figure 6:**
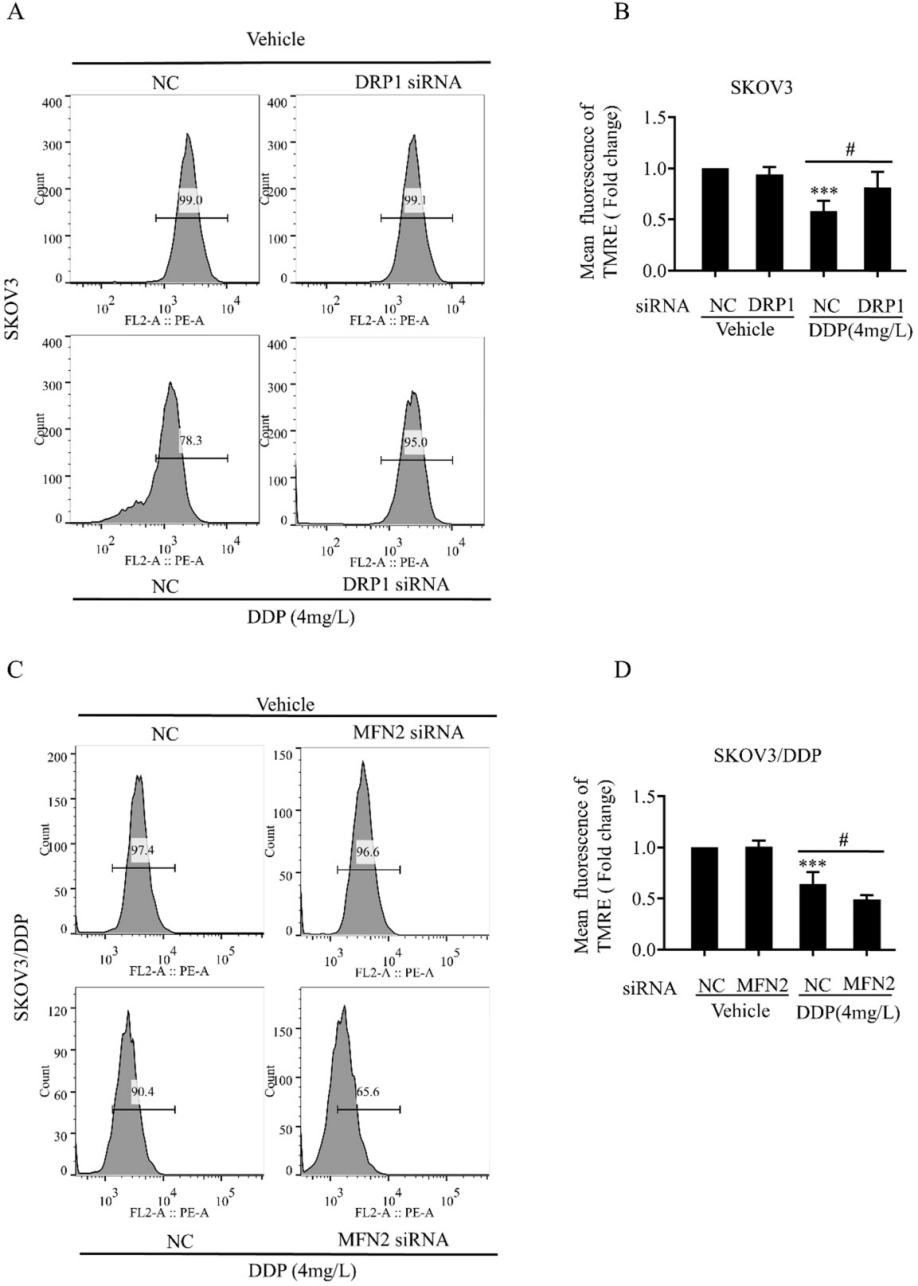
** Mitochondrial dynamics mediated by DRP1 and MFN2 regulates the level of mitochondrial membrane potential in SKOV3 and SKOV3/DDP cells after cisplatin.** (A, C) The effect of DRP1 siRNA and MFN2 siRNA on DDP-induced disruption of mitochondrial membrane potential in SKOV3/DDP cells. After the indicated treatments, cells were stained with TMRE to label mitochondrial membrane potential, and then mitochondrial membrane potential was evaluated by flow cytometry. (B, D) Ten thousand cells in each group were analyzed and the mean fluorescence intensity of TMRE in each group was measured from three independent experiments. ***p < 0.001 vs NC (Vehicle); ^#^p< 0.05.

**Figure 7:**
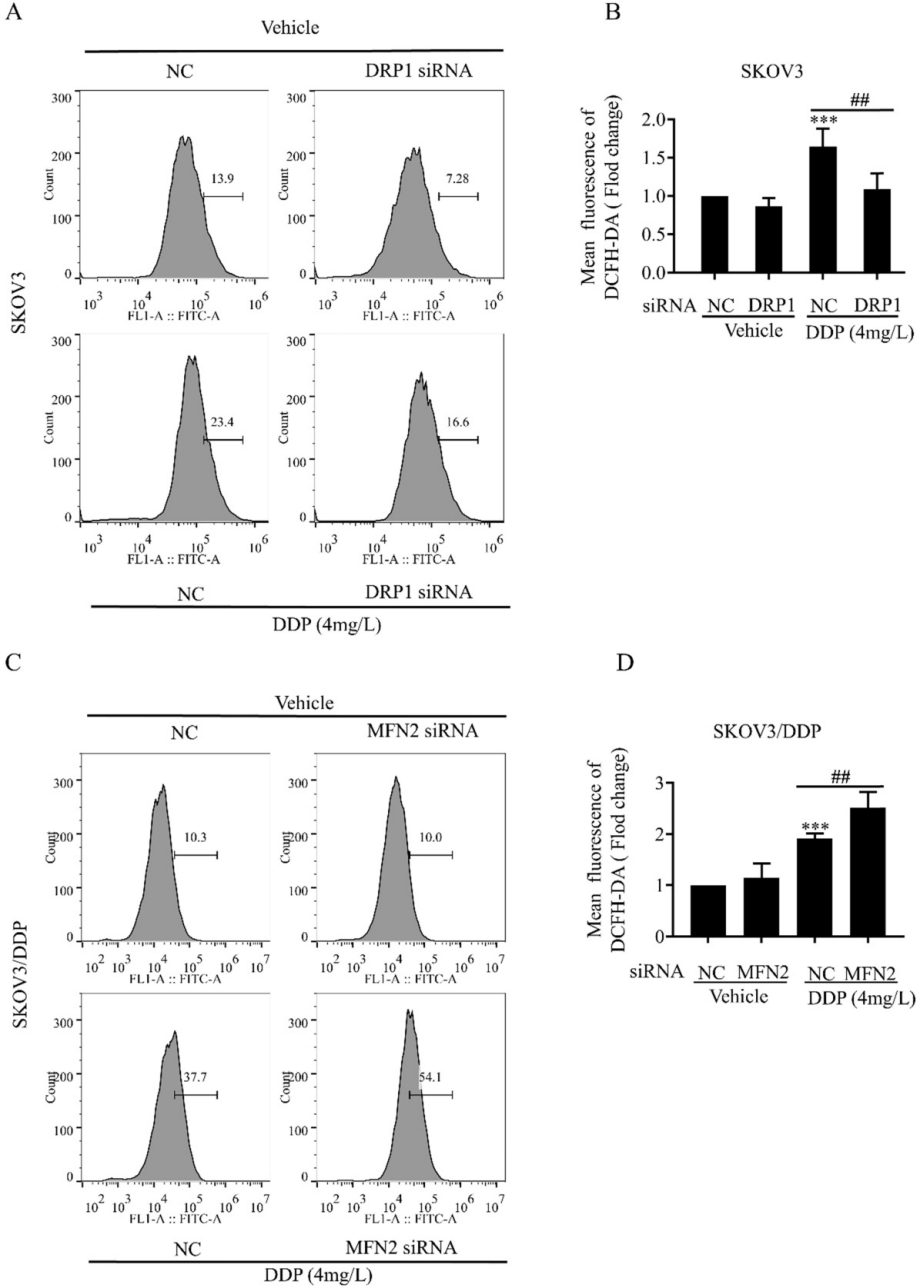
** Mitochondrial dynamics mediated by DRP1 and MFN2 regulates the intracellular ROS level in SKOV3 and SKOV3/DDP cells after cisplatin.** (A, C) Effect of mitochondrial dynamics mediated by DRP1 and MFN2 on DDP-induced intracellular ROS production in SKOV3 and SKOV3/DDP cells. After the indicated treatments, cells were stained with DCFH-DA, and then the intracellular ROS was measured by flow cytometry. (B, D) Ten thousand cells in each group were analyzed and the mean fluorescence intensity of DCFH-DA in each group was measured from three independent experiments. ***p < 0.001 vs NC (Vehicle); ^##^p< 0.01.

Mitochondrial dynamics are also important for the maintenance of mitochondrial functions including mitochondrial membrane potential and production of intracellular ROS 34. Next, we examined the role of DRP1 and MFN2-mediated mitochondrial dynamics in DDP-induced mitochondrial membrane potential and intracellular ROS. It was found that DRP1 siRNA effectively attenuated the DDP-induced disruption of mitochondrial membrane potential in SKOV3 cells (Fig. 6A and 6B), while MFN2 siRNA significantly aggravated DDP-induced disruption of mitochondrial membrane potential in SKOV3/DDP cells (Fig. 6C and 6D). Moreover, DRP1 siRNA significantly attenuated the DDP-induced intracellular ROS production in SKOV3 cells (Fig. 7A and 7B), while MFN2 siRNA markedly increased the DDP-induced intracellular ROS production in SKOV3/DDP cells (Fig. 7C and 7D). These results suggest that mitochondrial dynamics mediated by DRP1 and MFN2 may influence mitochondrial response to DDP in ovarian cancer cells.

### Mitochondrial dynamics mediated by DRP1 and MFN2 affects the migratory capability of SKOV3 and SKOV3/DDP cells

**Figure 8:**
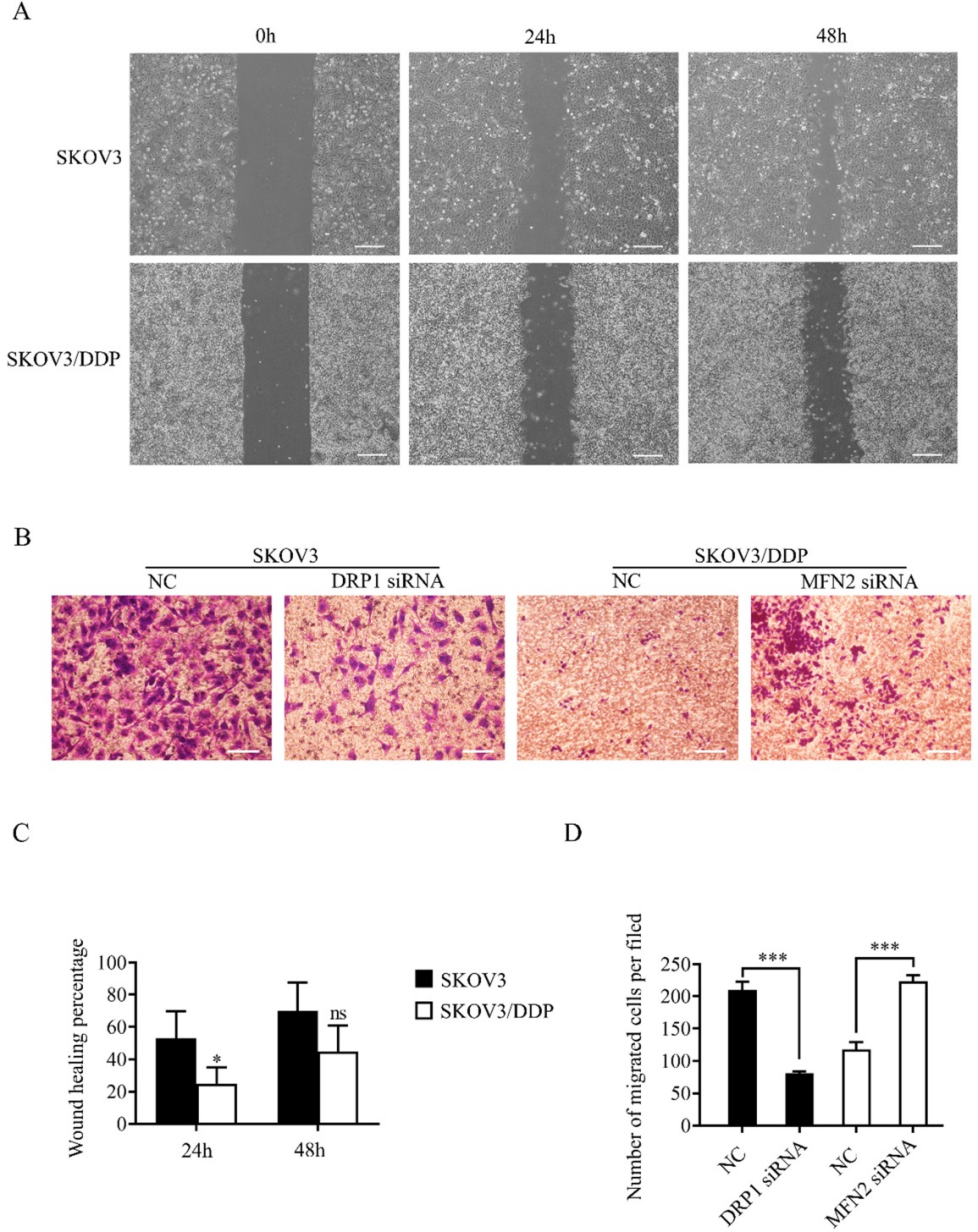
** Mitochondrial dynamics mediated by DRP1 and MFN2 regulates the migratory capability of SKOV3 and SKOV3/DDP cells.** (A, C) Migratory capability of SKOV3 and SKOV3/DDP cells was examined by wound healing assay. Cells in each group was photographed under a microscope at 0h, 24h, 48h after scratch, scale bar=400μm (A). The percent wound closure of each cell line at 24 h and 48 h after scratch was calculated from at least three fields in four independent experiments (C). (B) The migratory capability of SKOV3 and SKOV3/DDP was evaluated by Transwell assay. Scale bar=100μm. (D) The number of migrated cells in each group was calculated in three different fields from three independent experiments. *p < 0.05, ***p <0.001*.*

As mentioned above, mitochondrial dynamics mediated by DRP1 and MFN2 influence the level of ATP and ROS in ovarian cancer cells. Since cell migration is an energy-consuming process, and is also regulated by the increased intracellular ROS 35, we evaluated the migratory capability of SKOV3 and SKOV3/DDP cells using wound healing and transwell assay. The results of the wound healing assay showed the percent wound closure of SKOV3/DDP was significantly lower than that of SKOV3 at 24 h after scratch, although no significant difference between two cell lines was detected at 48 h (Fig. 8A and 8C). In transwell assay, the migrated SKOV3 cells were also more than the migrated SKOV3/DDP cells. Intriguingly, DRP1 siRNA significantly reduced the migrated SKOV3 cells, while MFN2 siRNA dramatically increased the number of migrated SKOV3/DDP cells (Fig. 8B and 8D). These results suggest that the mitochondrial dynamics mediated by DRP1 and MFN2 may affect the migratory capability of both SKOV3 and SKOV3/DDP cells.

### The mitochondrial dynamics mediated by DRP1 and MFN2 participates in DDP-induced intrinsic apoptosis pathway in SKOV3 and SKOV3/DDP cells

**Figure 9:**
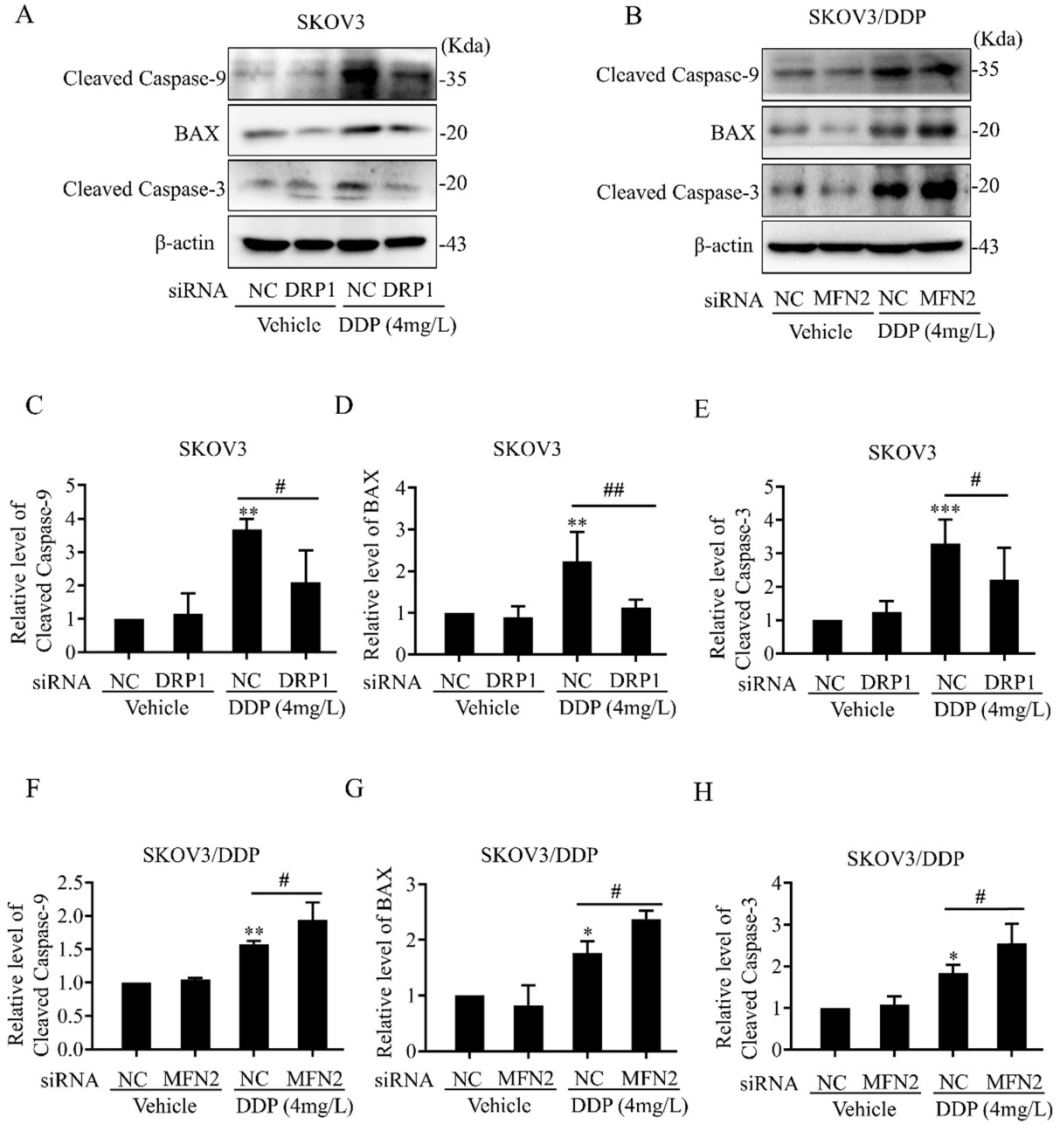
** Effects of mitochondrial dynamics mediated by DRP1 and MFN2 on DDP-induced intrinsic apoptosis pathway in SKOV3 and SKOV3/DDP cells.** (A, B) The expression of pro-apoptotic protein BAX and cleavage of Caspase-3/9 was examined by western blotting assay in SKOV3 and SKOV3/DDP cells. After the indicated treatments, the pro-apoptotic protein BAX and cleavage of Caspase-3/9 in each group were detected by immunoblotting, and β-actin was used as the endogenous reference. (C-H) The densitometric analysis of BAX and Cleaved Caspase-3/9 in (A) and (B) was performed from at least three independent experiments. The relative expression level of each protein is indicated as a normalization of the ratio of interest protein/β-actin in each sample to the control. * *p* < 0.05, ** p < 0.01, *** p < 0.001* vs. NC (Vehicle);* #* p* < 0.05*.*

Intracellular ROS, as an active second messenger, regulates a variety of cellular activities including oxidative stress and intrinsic apoptosis pathway (also known as mitochondrial apoptosis pathway) 36, 37. Mitochondrial dynamics mediated by DRP1 and MFN2 affects DDP-induced ROS production in SKOV3 and SKOV3/DDP cells (Fig. 7). Thus, we examined whether mitochondrial dynamics mediated by DRP1 and MFN2 contributes to DDP chemoresistance through inhibiting the intrinsic apoptosis pathway in ovarian cancer cells. First, the expression of intrinsic apoptosis-related proteins including BAX, Cleaved Caspase-3/9 was examined. As shown in Fig. 9A and 9B, 4 mg/L DDP increased the expression of pro-apoptotic protein BAX, cleavage of Caspase-3 and Caspase-9 in SKOV3 and SKOV3/DDP cells. Knockdown of DRP1 by siRNA significantly alleviated DDP-induced upregulation of BAX and cleavage of Caspase-3/9 in SKOV3 cells (Fig. 9C~9E). In contrast, silencing MFN2 markedly aggravated DDP-induced upregulation of BAX and cleavage of Caspase-3/9 in SKOV3/DDP cells (Fig. 9F~9H). These results suggest that mitochondrial dynamics mediated by DRP1 and MFN2 may contribute to DDP chemoresistance in ovarian cancer cells via participating in the intrinsic apoptosis pathway.

## Discussion

Ovarian cancer becomes the second leading cause of female gynecologic cancer death in the world 1. The high mortality of ovarian cancer is mainly due to the lack of early diagnosis and the development of chemoresistance 38. As the first line chemotherapeutic agent, DDP chemoresistance is the key cause of ovarian cancer recurrence, and is also a big challenge for the treatment of ovarian cancer 39. It is well documented that the increased DDP efflux, cytosolic inactivation of DDP and enhanced DNA repair are complicated in DDP chemoresistance 4, 5. In the present study, mitochondria, the target of DDP, were also found to participate in the development of DDP resistance in ovarian cancer cells. Human ovarian cancer cell line SKOV3 and its DDP-resistant cell line SKOV3/DDP were used in this study. As shown in Fig. 1A and 1B, SKOV3/DDP cells were more resistant to DDP, and the IC_50_ of DDP against SKOV3/DDP was also higher. Compared with parental SKOV3 cells, upregulation of MFN2 and downregulation of DRP1 were detected in the resistant SKOV3/DDP cells (Fig. 1C and 1D). Consistent with the expression profile of mitochondrial dynamics-related proteins, mitochondrial fusion was markedly enhanced in SKOV3/DDP cells (Fig. 1E and 1F). To explore the role of mitochondrial dynamics in the development of DDP resistance, the expression of mitochondrial dynamics-related proteins was further examined in SKOV3 cells under DDP stress. It was found that DDP stress induced downregulation of DRP1 and upregulation of all mitochondrial fusion proteins including MFN1, MFN2 and OPA1 (Fig. 2). However, the upregulation of MFN1 and OPA1 was not detected in the resistant SKOV3/DDP cells (Fig. 1). Although it is unclear why the expression profile of mitochondrial dynamics-related proteins was different between the resistant SKOV3/DDP cells and the parental SKOV3 cells under DDP stress, there are some explanations for this phenomenon. First, the parental SKOV3 cells were subjected to DDP stress for only 6 days, the short duration of DDP stress can only simulate the process of chemoresistance in a limited extent. As a result, there might be some differences between the resistant SKOV3/DDP and SKOV3 under DDP stress. Second, it is possible that mitochondrial fusion protein MFN1 and OPA1 also participate in the development of DDP resistance at the early stage, but are not involved in the maintenance of DDP resistance. Therefore, the consistent alterations of DRP1 and MFN2 in both SKOV3/DDP and SKOV3 under DDP stress strongly suggest its crucial role in the development of DDP chemoresistance in ovarian cancer. To further investigate the role of mitochondrial dynamics in the development of DDP resistance, knockdown of DRP1 or overexpressions of MFN2 were conducted in SKOV3 cells. It was found that knockdown of DRP1 increased the IC_50_ of DDP against SKOV3 cells from 1.41 mg/L to 2.98 mg/L, and overexpression of MFN2 increased the IC_50_ of DDP against SKOV3 cells to 4.54 mg/L (Fig.3C and Fig.3D). It seems that the effect of MFN2-mediated mitochondrial fusion is more evident than that of DRP1 siRNA. Thus, these results suggest that mitochondrial dynamics mediated by DRP1 and MFN2 may be involved in cisplatin resistance of ovarian cancer.

Mitochondria are the powerhouses providing most ATP for cells, and the high dynamic organelles as well 40. Mitochondrial dynamics plays an important role in maintenance of mitochondrial mass and function, and is also involved in a variety of cellular activities including proliferation, migration and apoptosis 41-43. Some large GTPases are identified as mediators of mitochondrial dynamics. Dynamin-related protein 1 (DRP1) and Fis1 regulate mitochondrial fission, while Mitofusion (MFN1, 2) and OPA1 mediate the fusion of mitochondrial outer and inner membrane, respectively 44-46. Herein, the enhanced mitochondrial fusion mediated by upregulated MFN2 and downregulated DRP1 were detected in the resistance SKOV3/DDP cells (Fig. 1E, 1F and Fig. 5). Compared with SKOV3 cells, the weaker migratory capability was detected in SKOV3/DDP cells. Intriguingly, knockdown of MFN2 in SKOV3/DDP cells increased the migration capability, while silencing DRP1 in SKOV3 cells had an opposite effect (Fig. 8). The role of mitochondrial dynamics mediated by DRP1 and MFN2 in migration of ovarian cancer cells is consistent with the previous studies 47, 48. It was reported that mitochondrial fission regulated the migration of malignant oncocytic thyroid tumor cells, breast cancer cells and lung cancer cells 49-51. DRP1-dependent mitochondrial fission was found to promote the migration of breast cancer cells through regulating the formation of lamellipodia 50. Moreover, NF-κB-inducing kinase also stimulates the migration of cancer cells via activating DRP1-dependent mitochondrial fission 52. However, the role of mitochondrial dynamics mediated by DRP1 and MFN2 in ATP level of ovarian cancer cells is contrary to the previous studies 47, 53. In this study, mitochondrial fusion mediated by upregulated MFN2 and downregulated DRP1 in SKOV3/DDP cells led to lower ATP level. Knockdown of MFN2 significantly increased the ATP level in SKOV3/DDP cells, although silencing DRP1 had no significant effect on the ATP level in SKOV3 cells (Fig. 5C). In agreement with this study, overexpression of MFN2 and knockdown of DRP1 in KPC cells also was reported to reduce oxidative phosphorylation and ATP production 54. Contrarily, lacking MFN1, 2, or the knockdown of OPA1 in mouse embryonic fibroblasts led to defects in multiple respiratory complexes, reduce oxidative phosphorylation and ATP production 55. In HeLa cells, silencing DRP1 induced a decrease in mitochondrial respiration and intracellular ATP level 56. In our opinion, it seems that the SKOV3/DDP cells are in a quiescent state with low ATP level and low migratory capability similar to cancer stem cells. Cancer stem cells are in a quiescent state with low proliferative capability and keep resistant to chemotherapy agents 57, 58. The quiescent state will be beneficial to the survival of the resistant SKOV3/DDP in extremely hard environments. Second, the different effects of mitochondrial dynamics on intracellular ATP generation might be derived from the cell context, although it remains to be elucidated. Anyway, our data and the previous studies suggest that the balanced mitochondrial fission and fusion are important for the maintenance of mitochondrial function and ATP generation.

Moreover, mitochondrial membrane potential is also important for the maintenance of mitochondrial function. Mitochondrial membrane potential derived from the electron transport chain facilitates the conversion of ADP to ATP 59. In this study, DDP treatment significantly reduced mitochondrial membrane potential in both SKOV3 and SKOV3/DDP cells. It is well known that excessive fission may lead to mitochondrial defects including disruption of mitochondrial membrane potential. Thus, knockdown of MFN2 aggravated the DDP-induced collapse of mitochondrial membrane potential in SKOV3/DDP cells, while silencing DRP1 attenuated the DDP-induced disruption of mitochondrial membrane potential in SKOV3 cells (Fig. 6). It also implied that mitochondrial dynamics mediated by DRP1 and MFN2 may be involved in the DDP-induced damage to electron transport chain in ovarian cancer cells. As the main source of intracellular ROS derives from the mitochondrial electron transport chain 60, the alteration of mitochondrial dynamics may influence the production of intracellular ROS. In this study, knockdown of MFN2 aggravated DDP-induced ROS in SKOV3/DDP cells, while silencing DRP1 alleviated DDP-induced ROS in SKOV3 cells (Fig. 7). ROS is an active second messenger, which participates in a variety of cellular activities including intrinsic apoptosis 37, inflammation signals 61, autophagy 62, and so on. High intracellular ROS inhibits the proliferation of cancer cells and induces the G2/M cell cycle arrest 63. The increased ROS production also sensitizes the advanced serous ovarian cancer to DDP 64. Consistent with the previous studies, knockdown of DRP1 in SKOV3 cells not only alleviated DDP-induced ROS production, but also decreased the expression of pro-apoptotic protein BAX and cleavage of Caspase-3 and Caspase-9 after DDP. In contrast, knockdown of MFN2 in SKOV3/DDP cells aggravated DDP-induced ROS production, and increased the expression of BAX and cleavage of Caspase-3 and Caspase-9 (Fig. 9). These results suggest that mitochondrial fission mediated by DRP1 facilitates the activation of the intrinsic apoptosis pathway after DDP, while mitochondrial fusion mediated by MFN2 promotes the DDP resistance in ovarian cancer cells through inhibiting the intrinsic apoptosis. However, it is still unclear how DRP1 is downregulated and MFN2 is upregulated in the resistant SKOV3/DDP cells. In our view, there are some clues for the underlying mechanism of how the expression of mitochondrial dynamics related proteins is changed in DDP resistant ovarian cancer cells. First, it was reported that some noncoding RNAs were involved in the DDP chemoresistance of ovarian cancer. For instance, miR-21-5p was found to directly downregulate the expression of DRP1^65^ and miR-21-3p facilitates resistance of ovarian cancer to platinum treatment 66. Thus, it cannot exclude the possibility that some noncoding RNAs can enhance the DDP chemoresistance in ovarian cancer cells through regulating the expression of mitochondrial dynamics related proteins. Second, atractylenolide III protects against cerebral ischemic injury through preventing neuroinflammation mediated by JAK2/STAT3/DRP1-dependent mitochondrial fission 67. This study implies the possible relationship between the transcription factor STAT3 and DRP1. Therefore, it will also be interesting to investigate whether DDP affects the activity of the transcription factor STAT3 and regulates the STAT3/DRP1 axis in DDP-resistant ovarian cancer cells.

**Figure 10:**
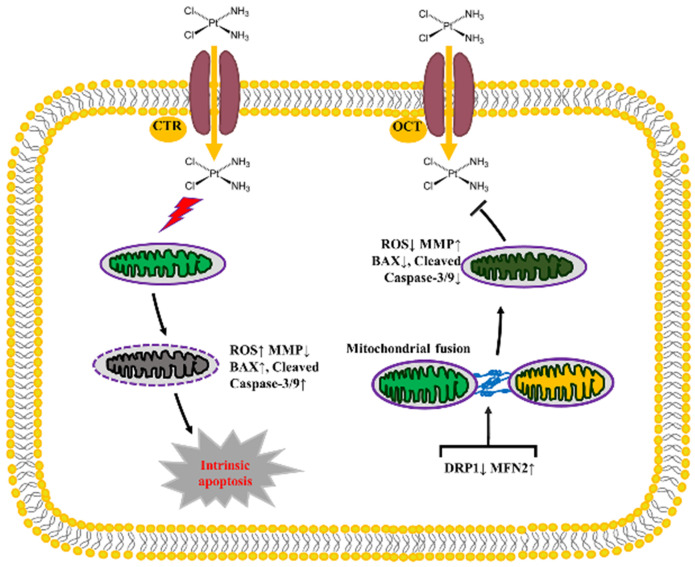
** The diagram showing the role of mitochondrial dynamics mediated by DRP1 and MFN2 in the DDP chemoresistance in ovarian cancer.** Cisplatin uptake into cells by copper influx transporter (CTR) or organic cation transporter (OCT) insults mitochondrial functions with enhanced ROS level, mitochondrial membrane potential (MMP) disruption and then triggers the intrinsic apoptosis of ovarian cancer cells by upregulating BAX and Cleaved Caspase-3/9. Downregulation of DRP1 and upregulation of MFN2 facilitate mitochondrial fusion, thereby preventing DDP-induced ROS production, MMP disruption and the intrinsic apoptosis, which promotes the chemoresistance of ovarian cancer to cisplatin.

In conclusion, our data in the present study indicate that the mitochondrial dynamics mediated by DRP1 and MFN2 is involved in the development of DDP chemoresistance in ovarian cancer through influencing the intracellular level of ATP and ROS, the disruption of mitochondrial membrane potential and intrinsic apoptosis (Fig. 10). Knockdown of DRP1 increases mitochondrial fusion, and facilitates the resistance of ovarian cancer cells to DDP. Silencing MFN2 stimulates mitochondrial fission, and sensitizes the resistant ovarian cancer cells to DDP. Therefore, it provides a new strategy to sensitize cancer cells to DDP chemotherapy or prevent DDP chemoresistance by targeting mitochondrial dynamics.

## Abbreviations

DDP: cisplatin
MMP: mitochondrial membrane potential
DRP1: dynamin-related protein 1
OPA1: optic atrophy 1
MFN1: mitofusin1
MFN2: mitofusin2
Fis1: mitochondrial adaptor fission1
ROS: reactive oxygen species

## References

[1] Lheureux S, Braunstein M, Oza AM (2019). Epithelial ovarian cancer: Evolution of management in the era of precision medicine. CA Cancer J Clin.

[2] Han XJ, Wei YF, Wan YY (2014). Development of a novel liposomal nanodelivery system for bioluminescence imaging and targeted drug delivery in ErbB2-overexpressing metastatic ovarian carcinoma. Int J Mol Med.

[3] Pinato DJ, Graham J, Gabra H (2013). Evolving concepts in the management of drug resistant ovarian cancer: dose dense chemotherapy and the reversal of clinical platinum resistance. Cancer Treat Rev.

[4] Harrach S, Ciarimboli G (2015). Role of transporters in the distribution of platinum-based drugs. Front Pharmacol.

[5] Oberoi HS, Nukolova NV, Kabanov AV (2013). Nanocarriers for delivery of platinum anticancer drugs. Adv Drug Deliv Rev.

[6] Galluzzi L, Senovilla L, Vitale I (2012). Molecular mechanisms of cisplatin resistance. Oncogene.

[7] Shoeib T, Sharp BL (2012). Interactions of oxaliplatin with the cytoplasmic thiol containing ligand glutathione. Metallomics.

[8] Martincuks A, Li PC, Zhao Q (2020). CD44 in Ovarian Cancer Progression and Therapy Resistance-A Critical Role for STAT3. Front Oncol.

[9] Ai Z, Lu Y, Qiu S (2016). Overcoming cisplatin resistance of ovarian cancer cells by targeting HIF-1-regulated cancer metabolism. Cancer Lett.

[10] Deng H, Ma J, Liu Y (2019). Combining alpha-Hederin with cisplatin increases the apoptosis of gastric cancer *in vivo* and *in vitro* via mitochondrial related apoptosis pathway. Biomed Pharmacother.

[11] Gualdani R, de Clippele M, Ratbi I (2019). Store-Operated Calcium Entry Contributes to Cisplatin-Induced Cell Death in Non-Small Cell Lung Carcinoma. Cancers (Basel).

[12] Chan DC (2020). Mitochondrial Dynamics and Its Involvement in Disease. Annu Rev Pathol.

[13] Archer SL (2013). Mitochondrial dynamics-mitochondrial fission and fusion in human diseases. N Engl J Med.

[14] Rovira-Llopis S, Banuls C, Diaz-Morales N (2017). Mitochondrial dynamics in type 2 diabetes: Pathophysiological implications. Redox Biol.

[15] Yu Y, Wang L, Delguste F (2017). Advanced glycation end products receptor RAGE controls myocardial dysfunction and oxidative stress in high-fat fed mice by sustaining mitochondrial dynamics and autophagy-lysosome pathway. Free Radic Biol Med.

[16] Yi X, Guo W, Shi Q (2019). SIRT3-Dependent Mitochondrial Dynamics Remodeling Contributes to Oxidative Stress-Induced Melanocyte Degeneration in Vitiligo. Theranostics.

[17] Zhang T, Wu P, Zhang JH (2018). Docosahexaenoic Acid Alleviates Oxidative Stress-Based Apoptosis Via Improving Mitochondrial Dynamics in Early Brain Injury After Subarachnoid Hemorrhage. Cell Mol Neurobiol.

[18] Pena-Blanco A, Garcia-Saez AJ (2018). Bax, Bak and beyond - mitochondrial performance in apoptosis. FEBS J.

[19] Morita M, Prudent J, Basu K (2017). mTOR Controls Mitochondrial Dynamics and Cell Survival via MTFP1. Mol Cell.

[20] van der Bliek AM, Shen Q, Kawajiri S (2013). Mechanisms of mitochondrial fission and fusion. Cold Spring Harb Perspect Biol.

[21] Yoo SM, Jung YK (2018). A Molecular Approach to Mitophagy and Mitochondrial Dynamics. Mol Cells.

[22] Chen M, Chen Z, Wang Y (2016). Mitophagy receptor FUNDC1 regulates mitochondrial dynamics and mitophagy. Autophagy.

[23] Liesa M, Shirihai OS (2013). Mitochondrial dynamics in the regulation of nutrient utilization and energy expenditure. Cell Metab.

[24] Ni HM, Williams JA, Ding WX (2015). Mitochondrial dynamics and mitochondrial quality control. Redox Biol.

[25] Grandemange S, Herzig S, Martinou JC (2009). Mitochondrial dynamics and cancer. Semin Cancer Biol.

[26] Han XJ, Yang ZJ, Jiang LP (2015). Mitochondrial dynamics regulates hypoxia-induced migration and antineoplastic activity of cisplatin in breast cancer cells. Int J Oncol.

[27] Han XJ, Shi SL, Wei YF (2017). Involvement of mitochondrial dynamics in the antineoplastic activity of cisplatin in murine leukemia L1210 cells. Oncol Rep.

[28] Li B, Wang W, Li Z (2017). MicroRNA-148a-3p enhances cisplatin cytotoxicity in gastric cancer through mitochondrial fission induction and cyto-protective autophagy suppression. Cancer Lett.

[29] Casinelli G, LaRosa J, Sharma M (2016). N-Myc overexpression increases cisplatin resistance in neuroblastoma via deregulation of mitochondrial dynamics. Cell Death Discov.

[30] Li JY, Zhang K, Xu D (2018). Mitochondrial Fission Is Required for Blue Light-Induced Apoptosis and Mitophagy in Retinal Neuronal R28 Cells. Front Mol Neurosci.

[31] Wan YY, Zhang JF, Yang ZJ (2014). Involvement of Drp1 in hypoxia-induced migration of human glioblastoma U251 cells. Oncol Rep.

[32] Balaban RS, Nemoto S, Finkel T (2005). Mitochondria, oxidants, and aging. Cell.

[33] Zorov DB, Juhaszova M, Sollott SJ (2014). Mitochondrial reactive oxygen species (ROS) and ROS-induced ROS release. Physiol Rev.

[34] Willems PH, Rossignol R, Dieteren CE (2015). Redox Homeostasis and Mitochondrial Dynamics. Cell Metab.

[35] Dong L, Lin T, Li W (2021). Antioxidative effects of polypyrimidine tract-binding protein-associated splicing factor against pathological retinal angiogenesis through promotion of mitochondrial function. J Mol Med (Berl).

[36] Sinha K, Das J, Pal PB (2013). Oxidative stress: the mitochondria-dependent and mitochondria-independent pathways of apoptosis. Arch Toxicol.

[37] Circu ML, Aw TY (2010). Reactive oxygen species, cellular redox systems, and apoptosis. Free Radic Biol Med.

[38] Khalifa AM, Elsheikh MA, Khalifa AM (2019). Current strategies for different paclitaxel-loaded Nano-delivery Systems towards therapeutic applications for ovarian carcinoma: A review article. J Control Release.

[39] van Zyl B, Tang D, Bowden NA (2018). Biomarkers of platinum resistance in ovarian cancer: what can we use to improve treatment. Endocr Relat Cancer.

[40] Madreiter-Sokolowski CT, Ramadani-Muja J, Ziomek G (2019). Tracking intra- and inter-organelle signaling of mitochondria. FEBS J.

[41] Chen H, Chan DC (2017). Mitochondrial Dynamics in Regulating the Unique Phenotypes of Cancer and Stem Cells. Cell Metab.

[42] Grumbach IM, Nguyen EK (2019). Metabolic Stress. Arterioscler Thromb Vasc Biol.

[43] Simula L, Nazio F, Campello S (2017). The mitochondrial dynamics in cancer and immune-surveillance. Semin Cancer Biol.

[44] Chen H, Detmer SA, Ewald AJ (2003). Mitofusins Mfn1 and Mfn2 coordinately regulate mitochondrial fusion and are essential for embryonic development. J Cell Biol.

[45] Cipolat S, Martins de Brito O, Dal Zilio B (2004). OPA1 requires mitofusin 1 to promote mitochondrial fusion. Proc Natl Acad Sci U S A.

[46] Frank S, Gaume B, Bergmann-Leitner ES (2001). The role of dynamin-related protein 1, a mediator of mitochondrial fission, in apoptosis. Dev Cell.

[47] Pan L, Zhou L, Yin W (2018). miR-125a induces apoptosis, metabolism disorder and migrationimpairment in pancreatic cancer cells by targeting Mfn2-related mitochondrial fission. Int J Oncol.

[48] You MH, Jeon MJ, Kim SR (2021). Mitofusin-2 modulates the epithelial to mesenchymal transition in thyroid cancer progression. Sci Rep.

[49] Ferreira-da-Silva A, Valacca C, Rios E (2015). Mitochondrial dynamics protein Drp1 is overexpressed in oncocytic thyroid tumors and regulates cancer cell migration. PLoS One.

[50] Zhao J, Zhang J, Yu M (2013). Mitochondrial dynamics regulates migration and invasion of breast cancer cells. Oncogene.

[51] Che TF, Lin CW, Wu YY (2015). Mitochondrial translocation of EGFR regulates mitochondria dynamics and promotes metastasis in NSCLC. Oncotarget.

[52] Jung JU, Ravi S, Lee DW (2016). NIK/MAP3K14 Regulates Mitochondrial Dynamics and Trafficking to Promote Cell Invasion. Curr Biol.

[53] Gao T, Zhang X, Zhao J (2020). SIK2 promotes reprogramming of glucose metabolism through PI3K/AKT/HIF-1alpha pathway and Drp1-mediated mitochondrial fission in ovarian cancer. Cancer Lett.

[54] Yu M, Nguyen ND, Huang Y (2019). Mitochondrial fusion exploits a therapeutic vulnerability of pancreatic cancer. JCI Insight.

[55] Chen H, Chomyn A, Chan DC (2005). Disruption of fusion results in mitochondrial heterogeneity and dysfunction. J Biol Chem.

[56] Parone PA, Da Cruz S, Tondera D (2008). Preventing mitochondrial fission impairs mitochondrial function and leads to loss of mitochondrial DNA. PLoS One.

[57] Dean M, Fojo T, Bates S (2005). Tumour stem cells and drug resistance. Nat Rev Cancer.

[58] Li L, Bhatia R (2011). Stem cell quiescence. Clin Cancer Res.

[59] Alpert NM, Guehl N, Ptaszek L (2018). Quantitative *in vivo* mapping of myocardial mitochondrial membrane potential. PLoS One.

[60] Rigoulet M, Yoboue ED, Devin A (2011). Mitochondrial ROS generation and its regulation: mechanisms involved in H(2)O(2) signaling. Antioxid Redox Signal.

[61] Blaser H, Dostert C, Mak TW (2016). TNF and ROS Crosstalk in Inflammation. Trends Cell Biol.

[62] Scherz-Shouval R, Elazar Z (2007). ROS, mitochondria and the regulation of autophagy. Trends Cell Biol.

[63] Greenshields AL, Shepherd TG, Hoskin DW (2017). Contribution of reactive oxygen species to ovarian cancer cell growth arrest and killing by the anti-malarial drug artesunate. Mol Carcinog.

[64] Kleih M, Bopple K, Dong M (2019). Direct impact of cisplatin on mitochondria induces ROS production that dictates cell fate of ovarian cancer cells. Cell Death Dis.

[65] Zhang JJ, Liu WQ, Peng JJ (2017). miR-21-5p/203a-3p promote ox-LDL-induced endothelial cell senescence through down-regulation of mitochondrial fission protein Drp1. Mech Ageing Dev.

[66] Pink RC, Samuel P, Massa D (2015). The passenger strand, miR-21-3p, plays a role in mediating cisplatin resistance in ovarian cancer cells. Gynecol Oncol.

[67] Zhou K, Chen J, Wu J (2019). Atractylenolide III ameliorates cerebral ischemic injury and neuroinflammation associated with inhibiting JAK2/STAT3/Drp1-dependent mitochondrial fission in microglia. Phytomedicine.

